# Pregnancy in Congenital Heart Disease, Complicated by Pulmonary Arterial Hypertension—A Challenging Issue for the Pregnant Woman, the Foetus, and Healthcare Professionals

**DOI:** 10.3390/medicina58040476

**Published:** 2022-03-25

**Authors:** Virginija Rudienė, Lina Kaplerienė, Dovilė Jančauskaitė, Emilija Meškėnė, Eglė Palevičiūtė, Monika Laukytė-Slėnienė, Diana Gasiūnaitė, Diana Ramašauskaitė, Elena Jurevičienė, Lina Gumbienė

**Affiliations:** 1 Clinic of Cardiac and Vascular Diseases, Faculty of Medicine, Institute of Clinical Medicine, Vilnius University, LT-08661 Vilnius, Lithuania; lina.kapleriene@santa.lt (L.K.); dovile.jancauskaite@santa.lt (D.J.); emilija.meskene@santa.lt (E.M.); egle.paleviciute@santa.lt (E.P.); monika.laukyte@santa.lt (M.L.-S.); lina.gumbiene@santa.lt (L.G.); 2 Faculty of Medicine, Vilnius University, LT-03101 Vilnius, Lithuania; diana.ramasauskaite@santa.lt (D.R.); elena.jureviciene@santa.lt (E.J.); 3 Centre of Anaesthesiology, Intensive Therapy and Pain Management, Faculty of Medicine, Vilnius University, LT-08661 Vilnius, Lithuania; diana.gasiunaite@santa.lt; 4 Centre of Obstetrics and Gynaecology, Faculty of Medicine, Vilnius University, LT-08661 Vilnius, Lithuania; 5 Centre of Pulmonology and Allergology, Faculty of Medicine, Vilnius University, LT-08661 Vilnius, Lithuania; 6 Centre of Heart and Chest Surgery, Faculty of Medicine, Vilnius University, LT-08661 Vilnius, Lithuania

**Keywords:** pulmonary arterial hypertension, congenital heart disease, pregnancy, rare diseases, multidisciplinary team

## Abstract

*Background and Objectives*: Pregnancy and delivery in patients with congenital heart disease (CHD) and pulmonary arterial hypertension (PAH) carry a very high risk for maternal and foetal complications and are contraindicated according to the guidelines. In the last decades, when an available modern PAH-targeted medication therapy and a new management concept improved patients’ well-being and survival, some PAH-CHD females decided to conceive. Of note, despite advanced treatment and modern healthcare system possibilities, dealing with pregnancy in a diverse PAH-CHD population is still challenging. The study aimed to share our experience with PAH-CHD pregnancies and discuss the risk assessment and current management of these patients with the combination of two rare diseases. *Materials and Methods*: The retrospective search of pulmonary hypertension and adult CHD registries in our hospital was performed, selecting all patients with CHD and PAH who conceived pregnancy from 2013 to 2021. Baseline demographic, clinical, and functional characteristics and clinical outcomes were collected. *Results*: Thirteen pregnancies in eight patients with PAH-CHD resulted in seven live births, three miscarriages, and three terminations. Five women were diagnosed with Eisenmenger syndrome (ES) and three with residual PAH after CHD repair. Before pregnancy, half of them were in WHO functional class III. Seven (87.5%) patients received targeted PAH treatment with sildenafil during pregnancy. In addition, the two most severe cases were administered with iloprost during peripartum. Three ES patients delivered preterm by Caesarean section under general anaesthesia. No neonatal mortality was reported. Maternal complications were observed in half of our cases. One patient died 12 days after the delivery in another hospital due to deterioration of heart failure. *Conclusions*: On the basis of our clinical experience, we conclude that pregnancy and delivery carry a high risk for maternal complications and should be avoided in women with PAH-CHD. The individualised approach of multidisciplinary care and appropriate monitoring are mandatory in reducing the risk of adverse outcomes.

## 1. Introduction

Pulmonary arterial hypertension (PAH) is a chronic and devastating disease characterised by progressive thickening of the walls of pulmonary arterioles, which trigger increased pulmonary arterial pressure and growing pulmonary vascular resistance, leading to right ventricular failure, severe complications, and death [1,2,3]. PAH could develop in congenital heart defects (CHD) with shunts [2,3,4,5]. PAH associated with CHD (PAH-CHD) is divided into four subgroups with different types of disease manifestation, management, and outcomes. The most common subgroups are postoperative PAH (residual or newly developed) and Eisenmenger syndrome (ES)—a severe PAH and cyanosis due to large, not repaired, and inoperable congenital shunt lesions [3,4,5,6]. 

In the last decades, the availability of modern PAH-targeted therapy and the new concept of management improved patients’ well-being and survival [3,4,7,8]. Therefore, some females with PAH-CHD decide to conceive delivery. In this case, comprehensive multidisciplinary management is mandatory for the best outcomes [9,10,11,12]. 

Mechanical compression by the expanding uterus, increase in sex hormones, the increase in circulatory volume, and cardiac output are the main physiological changes during pregnancy and the peripartum period, inducing synergistic stress, further aggravating PAH, and contributing to severe complications [9,13,14]. Moreover, the dramatic hypercoagulability of pregnancy increases the risk of pulmonary thromboembolism. Pregnancy with ES is at particular risk of increased right-to-left shunting due to decreased systemic vascular resistance, which results in increased hypoxia, pulmonary vasoconstriction, and right heart failure [13]. The highest risk is at the early peripartum period [7,13,14,15]. Thus, pregnancy and delivery with PAH carry a very high risk for maternal and foetal complications and mortality, and are contraindicated according to the guidelines of the European Society of Cardiology (ESC) [3,4,9]. 

We would like to share our multidisciplinary team experience with 13 PAH-CHD pregnancies and to discuss the risk assessment and current management strategies of these patients with a combination of two rare diseases.

## 2. Materials and Methods

### 2.1. Study Design

A retrospective analysis of our hospital database for pulmonary hypertension and adult CHD was performed. All consecutive patients with CHD and PAH who conceived pregnancy from 2013 to 2021 were included in this study (in 2013, the perinatal centre was established in our hospital and the pregnancy heart team started its activity).

The study was conducted following the Lithuanian national guidelines on human experimentation and the Declaration of Helsinki and approved by the Vilnius Regional Biomedical Research Ethics Committee (No. 158200-15-822-333; No. 2020/1-1182-669).

PAH was diagnosed according to criteria proposed in the ESC/European Respiratory Society (ERS) guidelines [3]: mean pulmonary artery pressure ≥ 25 mmHg at rest, pulmonary wedge pressure ≤ 15 mmHg, pulmonary vascular resistance (PVR) ≥ 3 Wood Units (WU) were found at right heart catheterisation (RHC), and other possible causes of pulmonary hypertension (PH) were excluded [3]. Our CHD cardiologists consulted all patients for a precise diagnosis. For the risk assessment, we used the PAH risk stratification score proposed by ESC/ERS [3] and ESC modified World Health Organisation (WHO) maternal risk for pregnancy classification [9]. The American Society of Anesthesiologists (ASA) physical status classification system (ASAPS) was applied before anaesthesia (https://www.asahq.org/standards-and-guidelines/asa-physical-status-classification-system, accessed on 1 December 2021).

Patients’ characteristics, such as age, WHO functional class (FC), modified WHO (mWHO) risk class (RC) of maternal cardiovascular risk, 6 min walking test (6MWT) distance, brain natriuretic peptide (BNP) levels, oxygen saturation, and prescribed medications, were evaluated at the first visit during pregnancy (baseline data) and repeated at the third trimester and at the last follow-up visit (till 1 December 2021) in our centre. Time and mode of delivery, anaesthetic management, and breastfeeding history were reviewed. We gathered the data regarding maternal cardiac (deterioration of heart failure (HF), arrhythmias, thromboembolism, death) and obstetric complications (preeclampsia, preterm delivery, infection, postpartum haemorrhage) and foetal and neonatal complications (miscarriage, intrauterine foetal demise, small birth weight for gestational age (SGA) or intrauterine growth restriction, CHD, neonatal death) during pregnancy and the peripartum period. 

### 2.2. Statistical Analysis

Due to high heterogenicity and small sample size, only descriptive statistical analysis data uses the following numerical characteristics for continuous variables (minimum and maximum values, mean and standard deviation). Qualitative data were provided as numbers and percentages.

## 3. Results

### 3.1. Baseline Data Characteristics

Thirteen pregnancies in eight females with PAH-CHD were found in our database from 2013 to 2021. The mean age during pregnancy was 27.2 (±3.2) years. Five patients were diagnosed with ES and three with residual PAH after CHD repair. Most of the patients (*n* = 5 of 8) were diagnosed with anatomical simple shunt lesions. The remaining three patients with complex CHD (single ventricle with transposition of the great arteries in two patients and atrioventricular septal defect in one) had Eisenmenger physiology. Patients’ characteristics are shown in Table 1 and Table 2. 

Both patients with single ventricle circulation (no. 2 and 6 in the tables) had mildly reduced ventricular ejection fraction. Right atrial enlargement (area > 18 cm^2^) was found in two patients (no. 5 and 8 in the tables) by echocardiography. A very high PVR (>12 WU) before pregnancy was found in six cases: in all with ES and one with postoperative PAH (no. 1, 2, 4–6, and 8 in the tables) on RHC. 

Most of the patients, 87.5% (*n* = 7), had pre-pregnancy counselling by CHD specialists and were advised to avoid pregnancy. Safe and reliable contraception was discussed as well. One patient was lost to follow-up and was referred to our CHD and PH competence centres at 12 weeks of gestation (WG). 

Half of the patients (50%, *n* = 4) were treated with specific PAH drugs before pregnancies: two with monotherapy and two with combination therapy (Table 1). Three patients were managed with embryotoxic endothelin receptor antagonists (ERA) bosentan or ambrisentan. One patient conceived with two additional embryotoxic medications: warfarin and amiodarone (no. 5 in the tables).

At the baseline, four patients were in WHO FC I-II and four in FC III. All patients were at WHO RC IV. Low oxygen saturation (mean 74 (57–89)%) at rest was observed in all Eisenmenger’s patients, measured by a pulse oximeter. Elevated BNP levels were found in three cases (no. 5, 6, and 8 in the tables). According to the PH risk stratification score, two patients (no. 3 and 7 in the tables) were in the low-risk profile [3].

### 3.2. Management during Pregnancy

During the first trimester, all patients were consulted by the Multidisciplinary Pregnancy and Heart Team (MPHT), and termination of pregnancy was recommended in all except one case (with mild postoperative PAH). Six women decided to proceed and were further managed by our MPHT consisting of adult CHD cardiologists, PH specialists, high-risk obstetricians, and dedicated anaesthesiologists. During pregnancy, all except one patient (*n* = 7, 87.5%) received targeted PAH treatment with a phosphodiesterase type 5 (PDE5) inhibitor (sildenafil). In addition, the two most severe cases were administered with a prostacyclin analogue (iloprost). Half of the patients (no. 2, 5, 6, and 8 in tables) were on beta-blockers and iron supplements. Two (no. 5 and 6 in tables) were treated with potassium-sparing diuretics. Two patients (no. 2 and 5 in tables) had hypothyroidism and were on levothyroxine. 

### 3.3. Pregnancy Outcomes, Delivery, and In-Hospital Management

Thirteen pregnancies in eight women resulted in seven live births, three miscarriages (at 6–7 WG), and three terminations of pregnancy (ToP) at 7–8 WG. Length of hospitalisation and type of anaesthesia are shown in Table 2. Only 53.8% of all pregnancies reached more than 20 WG. Detailed information is shown in Table 2. Due to the mother’s worsening condition, all three patients with ES were hospitalised and treated in-hospital 3–10 days before delivery. For all of them, preterm delivery was induced (at 27–34 WG). All deliveries were precisely planned and performed in cardiac surgery or obstetric settings at a tertiary care hospital. Neonatologists participated in MPHT meetings and deliveries. Mechanical support in cases of acute HF was prepared. Overall, two women had three vaginal deliveries, with regional anaesthesia (RA) in one case. Caesarean section (CS) was performed in four patients. In our hospital, all three CS were performed under general anaesthesia (GA, (the inhaled fentanyl and sevofluorane were used)) by the decision of the MPHT. One CS was performed under RA in another hospital. For the first four cases (Table 1), experienced cardiovascular and obstetric anaesthesiologists were involved. Epidural catheter placement was refused in three women: due to coagulation status in two and supra-systemic pulmonary artery pressure in one. All except one were managed with prophylactic doses of low molecular weight heparin (LMWH)) and sildenafil, and two with loop diuretics in the hospital before delivery. All ES patients needed oxygen therapy. Iloprost inhalations were added in two patients. 

After delivery, all women were treated in the intensive care unit (ICU) for a mean of 4.8 (1–7) days. In one most severe case (no. 2 in tables), haemodynamic treatment was supported with noradrenaline (for 40 h) and milrinone (for 51 h). She required blood transfusion due to postpartum haemorrhage as well. Iloprost was changed to oral ERA at discharge in both of our patients.

### 3.4. Maternal Complications and Outcomes

No maternal complications were observed in six pregnancy terminations. PAH therapy was restored in one patient; the rest of the women did not discontinue the treatment. 

Maternal cardiac complications occurred in four higher risk profile PAH pregnancies, which proceeded more than 20 WG. The deterioration of HF was found in three cases with ES. Maternal mortality occurred in one patient (7.7% of all pregnancies). An 18-year-old woman with severe postoperative PAH, FC III, died 12 days after the delivery (no. 8 in the tables). She was on treatment with sildenafil for four years before pregnancy and used to miss planned follow-up visits. Targeted therapy with inhaled iloprost was recommended due to worsening PAH and HF, but the patient refused. She did not make contact with a CHD/PH specialist and delivered a healthy son by CS with RA in another hospital. The patient’s status was considered stable, and she was discharged four days after CS without PH specialist consultation. On the fifth day at home, the patient suddenly deteriorated and died the same day in the ICU of the regional hospital from acute HF. 

Only one maternal obstetric complication (postpartum haemorrhage) was detected (no. 2 in the tables).

### 3.5. Fetal and Neonatal Outcomes

From 13 pregnancies, 6 (46%) foetuses were lost at a mean 6.8 (6–8) WG. Seven live new-borns were delivered at a mean of 34.7 weeks (27–40), four (57%) of them were preterm. All neonates survived. Only one case was small for gestational age. Patent ductus arteriosus (PDA) was diagnosed in two preterm neonates. One underwent PDA surgery at seven months of age. The detailed neonatal characteristics are given in Table 2. Four babies were breastfed from 4 to 14 months, one for less than two weeks (due to mother’s death). All children were growing well by the last follow-up visit.

### 3.6. Follow Up

Six PAH-CHD patients had regular follow-up visits in our PH centre after pregnancy. The mean follow-up period was 72.25 ± 13.14 (ranging from 55 to 85) months. PAH treatment escalation was needed by five out of seven patients. Diuretics were prescribed for two patients, and ERA were resumed in three patients after pregnancy. Four patients received oxygen therapy. 

One woman has lived abroad for the last two years but occasionally contacts our specialists by e-mail. Her clinical status started to deteriorate six years after delivery when she discontinued PAH-specific therapy (by her own decision), wherein haemoptysis appeared. However, treatment with dual therapy was restored, and she was stabilised. 

On the last follow-up visit, two patients with postoperative PAH were in FC I-II (and low PAH risk profile), and five patients with ES in FC III. The mean oxygen saturation in ES was 80.6 (72–84)% at rest and 65 (50–83)% during exercise. The mean distance of 6MWT was 449 (±51.6) meters. One patient had an elevated BNP level (no. 5 in Table 1) on follow-up. 

## 4. Discussion

In this article, we presented our MPHT last decade experience on managing 13 pregnancies in 8 patients with PAH-CHD. Huge progress has taken place in the field: improved knowledge of physiological and haemodynamical changes during pregnancy and delivery in these patients; establishment of specialised joint multi-speciality teams for adult CHD, PH, and high-risk pregnancy; and increased possibilities of intensive care units, neonatology, and PAH treatment. Fortunately, all our patients survived, except one who was non-compliant and decided to deliver in another setting. Knowledge and experience, strong MPHT, advanced treatment availability, and forward planning could be essential for good outcomes. Of note, despite advanced medical therapy and modern healthcare system possibilities, dealing with pregnancy in a diverse PAH-CHD population is still challenging.

### 4.1. Risk Assessment and Pre-Pregnancy Management

All women with CHD should receive pre-pregnancy counselling on the risk of maternal morbidity and mortality [4,9,16]. However, pregnant women with PAH have an extremely high risk of maternal mortality or severe morbidity (maternal cardiac event rate from 40 to 100%); therefore, pregnancy in PAH-CHD is contraindicated (mWHO class IV) [9].

The maternal mortality rate decreased significantly in PAH-CHD: from 30 to 50% in systematic review and case series published until 2000 [17,18] to 23–33% in reports and studies during 2009–2014 [15,19,20,21]. In publications of recently (2000–2018) managed pregnancies, mortality rate decreased to 3.6–6.8% [22,23,24,25]. Furthermore, in some highly experienced centres, all patients (45 PAH-CHD pregnancies) survived [26,27]. 

Effective and safe contraception should be advised in all PAH-CHD fertile age females [4,9,11], and termination of the pregnancy should be discussed if pregnancy occurs [4,9]. Hormone-releasing intrauterine systems are at first choice among reversible contraception methods. Possible vasovagal reactions and risk of infection during device insertion should be considered. Ethinyloestradiol-containing medications increase the risk of thrombosis [9,11]. Progesterone-based contraception may have reduced efficacy in women treated with bosentan [9]. Regrettably, by our practice, some PAH-CHD patients decline medical advice. Developed protocols regarding contraception, good access to family planning services [11,28], and active participation of nurses and all PH centres’ staff in encouraging such patients for pregnancy avoidance could improve compliance. 

On the other hand, individualised advice should be applied to women with PAH-CHD who consider becoming pregnant. There is a practice that low-risk profile patients with well-controlled PAH (PVR < 6 WU) are informed about risk for mother and offspring but are not discouraged against pregnancy very strongly [29,30]. Of note, this strategy could be applied successfully only at advanced PH centres, where modern PAH management is available. Furthermore, the centre’s experience in the management of such cases should be discussed [10].

Patients with Eisenmenger’s syndrome have a higher risk than those with repaired CHD or PAH associated with left-to-right shunts [24,30,31,32,33,34,35]. In pregnant ES patients, systemic vasodilation leads to an increase in right and left shunts and a decrease in pulmonary flow, resulting in higher cyanosis, reduced cardiac output, and paradoxical embolism [9].

As one of our cases with postoperative PAH-CHD showed, in some patients, the diagnosis of PAH-CHD might be missed until pregnancy. In the registry of pregnancy and cardiac disease (ROPAC), 12.5% of women were diagnosed with PAH-CHD only during pregnancy [22]. Risk assessment in such patients is challenging. We chose to overestimate the risk and decided to postpone exercise testing and RHC after the delivery. 

### 4.2. Management during Pregnancy and Delivery

Pregnant women with PAH-CHD should be cared for by an MPHT, including a PH expert, at an experienced high-risk pregnancy and heart centre. Even moderate PAH may worsen during pregnancy; therefore, these patients should be followed regularly and frequently, often weekly, in the third trimester [9]. 

The risk factors and the signs of serious complications are worsening of FC, high/increasing BNP levels, reduced right ventricle systolic function, and pericardial effusion detected on transthoracic echocardiography [24,36].

In our study, almost all patients (an exception was one mild PAH) were treated with specific PAH therapy during pregnancy. As pregnancy is a high-risk condition for PAH patients, advanced aggressive treatment of PAH should be considered when patients become pregnant [9,11,15]. Delayed initiation or escalation of PAH treatment is a risk factor of maternal mortality [15]. PDE5 inhibitors, parenteral epoprostenol, and selexipag are recommended. ERA could be associated with embryopathy and should be discontinued unless it significantly increases the risk of deterioration [9].

Interestingly, a small number of patients proceeded with ERA during pregnancy with good outcomes [15,22] or were discontinued gradually in early pregnancy [29]. Furthermore, some experts suggest that withdrawal of ERA treatment could escalate the irreversible worsening of PAH, and thus patients and their relatives should be informed and actively participate in this decision [15,36]. Treatment with calcium channel blockers, iloprost, and treprostinil could be continued or started as indicated. Epoprostenol is not available in our country, and therefore we used the other prostacyclin—inhaled iloprost. The first patients of our series were PAH treatment-naïve before pregnancy (due to reimbursement difficulties at that time), but treatment was started immediately. Restricted access to expensive PAH therapy in some countries reflects recent systematic review data. In cohorts published between 2008 and 2018, less than half of the pregnant patients were managed with PAH targeted therapy, and maternal mortality was 11% in the PAH-CHD group [37]. Similar data were shown in the study from a tertiary centre in South India: only in 21.5% of 65 PAH-CHD patients was sildenafil administered, and 20% of cases with ES died [38].

Anticoagulation therapy should be continued if there was an indication before pregnancy. In the rest of the pregnant patients with PAH-CHD, decisions should be made individually, considering the risk of bleeding (which likely outweighs the benefits in ES patients) [9,11,23,36]. However, pulmonary thrombosis was one of the reasons for maternal mortality in patients with PAH-CHD without thromboprophylaxis [15]. In our study, one patient was on warfarin, and five were managed with a low dose of LMWH during hospitalisation. 

Furthermore, the need for oxygen therapy, diuretics, and iron replacement should be considered. 

On the basis of the data from a prospective multicentre ROPAC registry [39], the most common complications in patients with the PAH-CHD group were HF during pregnancy (31%) and ventricular arrhythmias (4.8%). Maternal mortality was 4.8%. The main causes of death for pregnant women with PAH-CHD are worsening of the right HF, pulmonary hypertensive crisis, pulmonary thrombosis, sepsis, and sudden death [9,15,24,37]. The highest rate of severe maternal complications (including death) was found in ES patients (66.7%) and patients with repaired defects (46.2%) [24]. The deterioration of HF developed in all our severe PAH-CHD cases after 20 WG. CHD patients with PH could have more obstetric events, including hypertension and preeclampsia [24,40,41]. The only obstetric complication was a postpartum haemorrhage in one patient from our cohort. 

The optimal timing and mode of delivery for women with PAH-CHD are objects to debate and should be decided individually by the pregnancy heart team. Late hospitalisation was found as a risk factor for maternal mortality [15] and planned early delivery at 32–36 weeks before maternal deterioration may improve the outcomes [10,11,21,36]. The postpartum need for intensive care and mechanical support should be discussed in advance [9]. 

Planned CS is recommended in severe forms of PAH-CHD, including ES [9,10], and has an advantage in the precise preparation of anaesthesia, optimisation of haemodynamics, and no need for maternal physical effort during labour [42]. Vaginal delivery is preferable in milder PAH-CHD forms as it is associated with slower rearrangement of haemodynamics after delivery as well as fewer complications (bleeding, thrombosis, infection), but increased risk of cardiovascular collapse during Valsalva manoeuvre [11] and emergency CS delivery [9,36]. 

These recommendations were successfully applied to our patients. 

Choice of anaesthesia (general or regional) is another challenge. Systematic analysis of the Brompton team revealed a fourfold increased risk of maternal mortality when GA was performed [15]. Emergency CS was often performed under GA in severe PAH-CHD patients [24,33], and the impact for mortality could be overestimated [11,15]. Regional anaesthesia is usually preferable in obstetrics and has an advantage to the neonate. Epidural anaesthesia provides excellent analgesia but could increase peripheral vasodilation and heart rate, leading to an increase in cardiac output [43]. Severe PAH-CHD patients can hardly deal with such an additional load [11]. Furthermore, the main limitations of applying regional anaesthesia are hypocoagulation and thrombocytopenia [44], as was observed in two of our patients, and GA was chosen. Both modes of anaesthesia have adverse effects for a parturient with PAH-CHD and should therefore be anticipated, prevented, and rapidly treated by the anaesthesiologist [9]. In some cases, GA is the only option, and nowadays, it can be safe [45], as shown by our experience and published successful cases [30,46]. 

Precise fluid balance, optimisation of right ventricle function, maintenance of oxygenation, and very close follow-up is mandatory after delivery [9,26,27,36].

### 4.3. The Outcomes

Early ToP is common in PAH patients with a rate of 21.7% and maternal mortality of 11% by the data of the recent systematic review [37]. In our historical data from 1967 to 2003, we observed an even higher rate of ToP—43%, but without maternal mortality [47]. A number of ToP deceased nearly twice (23%) in our present study, likely due to improved pre-pregnancy counselling and contraception advice. Indeed, it is still very high. 

The rate of spontaneous miscarriages was high (23.1%) in our cohort as in other PAH-CHD studies and referred up to 41% [23,27,37]. The severity of prenatal maternal hypoxemia predicted foetal outcomes [9,48]. It could be a possible reason for the high foetal loss rate in our ES patients. 

In our study, the mortality rate of PAH-CHD pregnancies was 7.7%, which is probably not reliable due to the limited sample size. Interestingly, in our former study (pregnancies in ES during 1967–2003) [47], we observed an even lower mortality rate (from 30 pregnancies and 13 deliveries in 10 women—1 maternal death three days after SC at 36 WG in other institution). Better FC (9 pts in FC II) and simple CHD (ventricular septal defects and PDA in all except one) should be taken into consideration.

The risk of maternal mortality is highest in the early peripartum and 1–3 months postpartum [9,11,15,24,37]. Our patient also died in the early postnatal period (8 days after delivery). Patient compliance, access to care and quality of healthcare were predictors of adverse events in pregnant women with heart disease in the CARPREG II study [49]. 

Intrauterine growth restriction was reported in 24–36% of neonates according to metanalyses of pregnancies in women with PAH [15,37]. A study of 11 PAH treatment-naive pregnancies with ES from China found SGA in 83% of new-borns [33]. Maternal treatment with PDE inhibitors could prevent SGA by improving the uteroplacental blood flow [50,51]. Moreover, our series and other published data [52] suggested that improving maternal status during pregnancy with intensive PAH therapy should be essential in assuring foetal growth. 

It is challenging to evaluate the impact of pregnancy on the long-term prognosis of PAH-CHD patients [15]. Most of our females (5 from 7) had clinical worsening at midterm follow up (mean time six years) after pregnancies, and PAH treatment was intensified. Similar data were published for the Hanover team [29]. Only 3 of 10 of our Eisenmenger patients [47] survived 17–30 years after delivery. All of them were on combination PAH therapy; one became a grandmother. 

## 5. Study Limitation

Given the retrospective nature of data analysis, some of the values were missing, which might have influenced the study results. Due to the small size of included cases and the lack of data, only descriptive statistics were used to describe the basic features of the data in a study. However, not all patients followed the recommendations and missed the follow-up period. Multicentre and prospective studies are required. 

## 6. Conclusions

Our present case series of pregnancy in PAH-CHD patients showed a big heterogenicity according to risk, course, and outcome. Even now, with new PAH treatment possibilities and advanced multidisciplinary high-risk pregnancy management concepts, results could be hardly predictable. The maternal and foetal complications rate is still high, and thus pregnancy should be discouraged in women with PAH-CHD.

## Figures and Tables

**Table 1:** Summary of patients’ baseline characteristics, management, and treatment.

Pts No.	Diagnosis (Procedures (at Age of))	FC	Pregnancy; Delivery/ Age (Years)	SpO2 (%) before Pregnancy/III Trimester/at Last Follow-Up	Hgb (G/L) before Pregnancy/ III Trimester/Last Follow-Up	BNP (ng/L) before Pregnancy/III Trimester/at Last Follow-Up	6MWT (M) before/during Pregnancy/ at Last Follow-Up	PAH Targeted Treatment during Pregnancy/Last Follow-Up	Duration of Breastfeeding (Months)
1	VSD, ES	II–III	1; 1 25 y.o.	82/86/84	177/165/161	<10/28/<10	420/360/430	Sildenafil (from II trimester)/Sildenafil and Bosentan	No
2	SV, TGA, ES	III	1; 1 35 y.o.	57/63/80	198/183/191	76/265/60	469/480/480	Sildenafil (from II trimester) + inhaled Iloprost (at early peripartum period)/Sildenafil, Ambrisentan	4 months
3	PDA (surgical ligation (5 years)), residual PAH	II	3; 2 31 y.o.	-/94/99	-/132/140	-/45/18	-/360/550	Sildenafil (from II trimester)/Sildenafil	12 months
4	VSD, PDA, ES	III	1; 0 26 y.o.	78/-/83	175/-/163	37/-/13	460/480/510	Sildenafil	-
			2; 1 28 y.o.	89/-/82	177/135/162	13/28/27	515/450/405	Sildenafil + Iloprost (at early peripartum period)/Sildenafil +, Bosentan (after delivery)	No
5	AVSD, ES	III	1; 0 24 y.o.	80/-/74	178/-/175	18/-/17	470/-/480	Ambrisentan	
			2; 0 26 y.o.	79/-/72	206/-/183	72/-/202	410/420/420	Ambrisentan	
			3; 0 27 y.o.	72/-/82	183/-/216	202/-/167	420/-/408	Ambrisentan	
6	SV, TGA, ES	III–IV	1; 0 23 y.o.	74/-/75	192/-/177	228/-/45	400/-/420	Sildenafil, Bosentan	-
			2; 0 29 y.o.	66/-/75	164/-/214	40/-/29	420/-/-	Sildenafil/Sildenafil + Bosentan	-
7	VSD (surgical closure (4 years)), CoA surgical correction (2 months), re-CoA balloon angioplasty (17 years), residual PAH	II	1; 1 30 y.o.	94/-/-	127/-/126	-/173/-	-/-/-	None	12 months
			2; 2 32 y.o.	-/-/-	126/-/118	/81/-	-/-/-	None	14 months
8	VSD (surgical closure (4 years), residual PAH	II	1; 1 18 y.o.	95/99/-	134/103/118	161/223/-	530/480/-	Sildenafil/ *	8 days

CoA—coarctation of aorta; ES—Eisenmenger syndrome; PAH—pulmonary arterial hypertension; PDA—patent ductus arteriosus; re-CoA—re-coarctation of aorta; SV—single ventricle; TGA—transposition of the great arteries; VSD—ventricular septal defect; FC—World Health Organisation functional class; SpO2—oxygen saturation; HgB—haemoglobin; BNP—B-type natriuretic peptide; 6MWT—six-minute walk test. * Unknown.

**Table 2:** The basic characteristic of pregnancy outcomes, delivery, and in-hospital management.

Patient	Age/Diagnosis	FC	ASA	Pregnancy; Delivery/ Mode of Delivery or ToP/Weeks of Gestation	Anaesthesia Type	Newborn Weight, High, Apgar Score	Hospitalisation/ICU or PACU Time (Days)
1	25 y.o. ES	III	IV	1; 1/CS/34 WG	GA	2160 g, 49 cm, Apgar 8/9	6 days ICU—3 days
2	35 y.o. ES	IV	IV	1; 1/CS/32 WG	GA	1400 g, 40 cm, Apgar 9/10	22 days ICU—7 days
3	31 y.o. Residual PH	II	IIIE	3; 2/VD/36 WG	RA (EA)	2790 g, 49 cm, Apgar 9/9	8 days ICU—4 days
4	26 y.o. ES	III	III	1; 0/missed abortion /6 WG	IV	-	2 days ICU—1 day
	28 y.o. ES	III	IV	2; 1/CS/27 WG	GA	900 g, 33 cm, Apgar 8/9	19 days ICU—7 days
5	24 y.o. ES	III	-	1; 0/miscarriage/6 WG	-	-	-
	26 y.o. ES	III	III	2; 0/missed abortion/7 WG	IV	-	5 days ICU—1 day
	27 y.o. ES	III	IV	3; 0/ToP/8 WG	IV	-	1 day PACU—1 h
6	23 y.o. ES	III	III	1; 0/ToP/7 WG	IV	-	7 days ICU—1 day
	29 y.o. ES	III	IIIE	2; 0/ToP/7 WG	IV	-	1 day PACU—1 h
7	30 y.o. Residual PH	I	-	1; 1/VD/40 WG	No anaesthesiological intervention	2960 g, 51 cm, Apgar 8/9	3 days
	32 y.o. Residual PH	I	-	2; 2/VD/37 WG	No anaesthesiological intervention	3240 g, 51 cm, Apgar 8/9	4 days
8	18 y.o. Residual PH	III/IV	*	1; 1/CS/37 WG	RA (EA)	2610 g, 48 cm, Apgar 9/9	5 days ICU—1 day

ASA—American Society of Anesthesiologists (ASA) physical status classification; GA—general anaesthesia; RA—regional anaesthesia; EA—epidural anaesthesia; IV—intravenous; PACU—post-anaesthesia care unit; ES—Eisenmenger syndrome; PH—pulmonary hypertension; CS—Caesarean section; FC—functional class; WG—weeks of gestation; y.o.—years old; ToP—termination of pregnancy; VD—vaginal delivery; Apgar score is a scoring system to assess new-borns one minute and five minutes after delivery. The Apgar score is based on a total score 1 of 10. * data not available, delivery in other hospital.

## Data Availability

Not applicable.
